# Methodological approaches for assessing certainty of the evidence in umbrella reviews: A scoping review

**DOI:** 10.1371/journal.pone.0269009

**Published:** 2022-06-08

**Authors:** Saranrat Sadoyu, Kaniz Afroz Tanni, Nontaporn Punrum, Sobhon Paengtrai, Warittakorn Kategaew, Nattiwat Promchit, Nai Ming Lai, Ammarin Thakkinstian, Surachat Ngorsuraches, Mukdarut Bangpan, Sajesh Veettil, Nathorn Chaiyakunapruk

**Affiliations:** 1 Pakchongnana Hospital, Nakhon Ratchasima, Thailand; 2 Department of Health Outcomes Research and Policy, Harrison College of Pharmacy, Auburn University, Auburn, Alabama, United States of America; 3 Faculty of Pharmacy, Chiangmai University, Chiang Mai, Thailand; 4 Department of Pharmacotherapy, College of Pharmacy, University of Utah, Salt Lake City, Utah, United States of America; 5 School of Medicine, Taylor’s University, Subang Jaya, Malaysia; 6 School of Pharmacy, Monash University Malaysia, Bandar Sunway, Malaysia; 7 Department of Clinical Epidemiology and Biostatistics, Faculty of Medicine, Ramathibodi Hospital, Mahidol University, Bangkok, Thailand; 8 Mahidol University Health Technology Assessment Graduate Program, Bangkok, Thailand; 9 The Evidence for Policy and Practice Information and Co-ordinating Centre (EPPI-Centre), Social Research Institute, University College London, London, United Kingdom; 10 IDEAS Center, Veterans Affairs Salt Lake City Healthcare System, Salt Lake City, Utah, United States of America; Xiamen University - Malaysia Campus: Xiamen University - Malaysia, MALAYSIA

## Abstract

**Introduction:**

The number of umbrella reviews (URs) that compiled systematic reviews and meta-analysis (SR-MAs) has increased dramatically over recent years. No formal guidance for assessing the certainty of evidence in URs of meta-analyses exists nowadays. URs of non-interventional studies help establish evidence linking exposure to certain health outcomes in a population. This study aims to identify and describe the methodological approaches for assessing the certainty of the evidence in published URs of non-interventions.

**Methods:**

We searched from 3 databases including PubMed, Embase, and The Cochrane Library from May 2010 to September 2021. We included URs that included SR-MAs of studies with non-interventions. Two independent reviewers screened and extracted data. We compared URs characteristics stratified by publication year, journal ranking, journal impact factor using Chi-square test.

**Results:**

Ninety-nine URs have been included. Most were SR-MAs of observational studies evaluating association of non-modifiable risk factors with some outcomes. Only half (56.6%) of the included URs assessed the certainty of the evidence. The most frequently used criteria is credibility assessment (80.4%), followed by GRADE approach (14.3%). URs published in journals with higher journal impact factor assessed certainty of evidence than URs published in lower impact group (77.1 versus 37.2% respectively, p < 0.05). However, criteria for credibility assessment used in four of the seven URs that were published in top ranking journals were slightly varied.

**Conclusions:**

Half of URs of MAs of non-interventional studies have assessed the certainty of the evidence, in which criteria for credibility assessment was the commonly used method. Guidance and standards are required to ensure the methodological rigor and consistency of certainty of evidence assessment for URs.

## Introduction

The number of systematic review (SR) and umbrella reviews (UR) and meta-analysis (MAs) has increased dramatically over recent years [1]. Most SRs and MAs focus on answering a question such as the effect of a single treatment comparison on an outcome, this leaves a big gap of lacking an overall summary of evidence addressing broader related questions. URs, also known as overview or review of reviews, evolved in the last decade, can summarize and even synthesize the findings into single comprehensive evidence answering the broader picture of all existing findings [1, 2]. As a result, URs are considered as one of the highest levels of evidence summary in biomedical literature [2]. URs of epidemiological investigations and non-interventional studies help establish evidence linking exposure to certain health outcomes in a population. Therefore, these studies are expected to play a key role in gauging the burden of diseases, understand the risk or protective factors, delineating guideline for prevention as well as streamlining the treatment development process [3, 4].

Some steps for performing URs are generally similar to SR-MAs (e.g., search strategy, study selection, data extraction), yet some others especially assessment of uncertainty methods are not applicable owing to the difference in types of included studies [5]. Recently, there is a methodological guidance focusing on conducting and reporting an UR [6]. However, it does not cover every single aspect of the process including assessment methods for certainty of the evidence.

The certainty of the evidence from URs is an essential component as it demonstrates the confidence of the findings found across studies leading to support a decision or recommendations. Several approaches have been used in literature. For instance, some URs adopted Grading of Recommendation, Assessment, Development and Evaluation (GRADE) approach, which was originally designed for assessing the certainty of evidence of primary studies included in SRs, not URs [7]. In contrast, some URs reported the certainty of included SRs and MAs as originally reported from each study without further assessment [8–10]. Furthermore, the UR of MAs is more challenging as they usually report summary statistical data as one of the objective criteria to grade the certainty of evidence. Recently, the relatively strict criteria for stratifying the certainty of evidence using several statistical parameters (i.e., degree of statistical significance, predictive interval, small-study effects, and excess significance bias) have also been used and suggested as the good practical tips for conducting good URs [2]. Currently, there is no formal guidance for assessing the certainty of evidence in URs. Therefore, this scoping review aims to identify and describe the methodological approaches for assessing the certainty of the evidence in published URs that included MAs of non-interventions.

## Methods

This review was conducted according to the methods pre-specified in a registered protocol (PROSPERO registration: CRD42020203273), following the Preferred Reporting Items for Systematic Reviews and Meta-Analyses (PRISMA) extension for scoping reviews (**S1 Table in** S1 File).

### Search strategy and selection criteria

We searched three databases including PubMed, Embase, and The Cochrane Library from May 2010 to Sep 2021. The keyword ‘umbrella review’ was used. The full search strategies without language restriction are described in **S2 Table in** S1 File. Manual searches of the reference lists of the eligible articles were also performed. We defined an UR as the review that is designed to summarize the evidence from multiple SR-MAs that were labeled as ‘umbrella review’ in the title or abstract of the article.

At least two reviewers (SS, KT, NP, SN, WK, NP, and SP) independently reviewed the titles, abstracts, and full texts for their potential inclusion against the eligibility criteria. Any disagreement was resolved by consensus with a third reviewer (NC). No language restriction was applied. URs, overview of SR-MAs, review of SR-MAs were selected if they included (a) MAs of interventions and (b) MAs of non-interventions involving diagnostic/prognostic factors and non-modifiable risk factors of diseases or health conditions, disease etiology, prevalence or incidence; in which most studies were observational studies, e.g., cohort, case-control, and cross-sectional studies. In this current study, we focus only umbrella reviews that included MAs of non-intervention studies.

Other types of studies or reviews (e.g., handbooks, guidelines, commentaries, editorials, and methodological studies), materials for poster presentations, UR of SRs without MAs, and protocols of URs were excluded. URs with network MAs were also excluded because these studies might use different methodological approaches.

### Data extraction

At least two reviewers (SS, NP, SN, KT, WK, MP, and SP) independently extracted the data from each UR into a customized data extraction table. Any disagreement was resolved by consensus with a third reviewer (NC). Details of data extraction are described in S1 File.

The assessment of the certainty of evidence was defined as any of evaluation of the totality or strength of the evidence such as the GRADE approach, criteria for credibility assessment, and other approaches used to grade the overall body of the UR evidence.

### Data synthesis and analysis

A descriptive analysis of the methodological approaches for assessing the certainty of evidence in the URs was performed by frequencies and percentage. The included URs were classified into high and low impact sources based on the journal impact factors (JIF) reported by the Institute of Scientific Information’s Journals Citation Report in 2021 accessed on October 21, 2021. The journals reported as the top 100 highest ranking were defined as the high, otherwise they were classified as low impact groups. In addition, we also classified based on a median JIF, i.e., high if the URs were published in JIF ≥ median, otherwise the URs were classified as lower impact groups. According to the previous study [11] that suggested the usefulness of URs according to the higher number of citations after 2015 and the release of tools for the methodological quality assessment in 2016 [12]. Thus, we further compared URs published between 2010 to 2016 with those published from 2017 to 2021, when feasible. Chi-square or Fisher’s exact test where appropriated was applied to compare characteristics of URs between groups. All analyses were performed using STATA version 15.0 (College Station, TX), p-value ≤ 0.05 was considered as statistical significance.

## Results

### Search results

**Fig 1:**
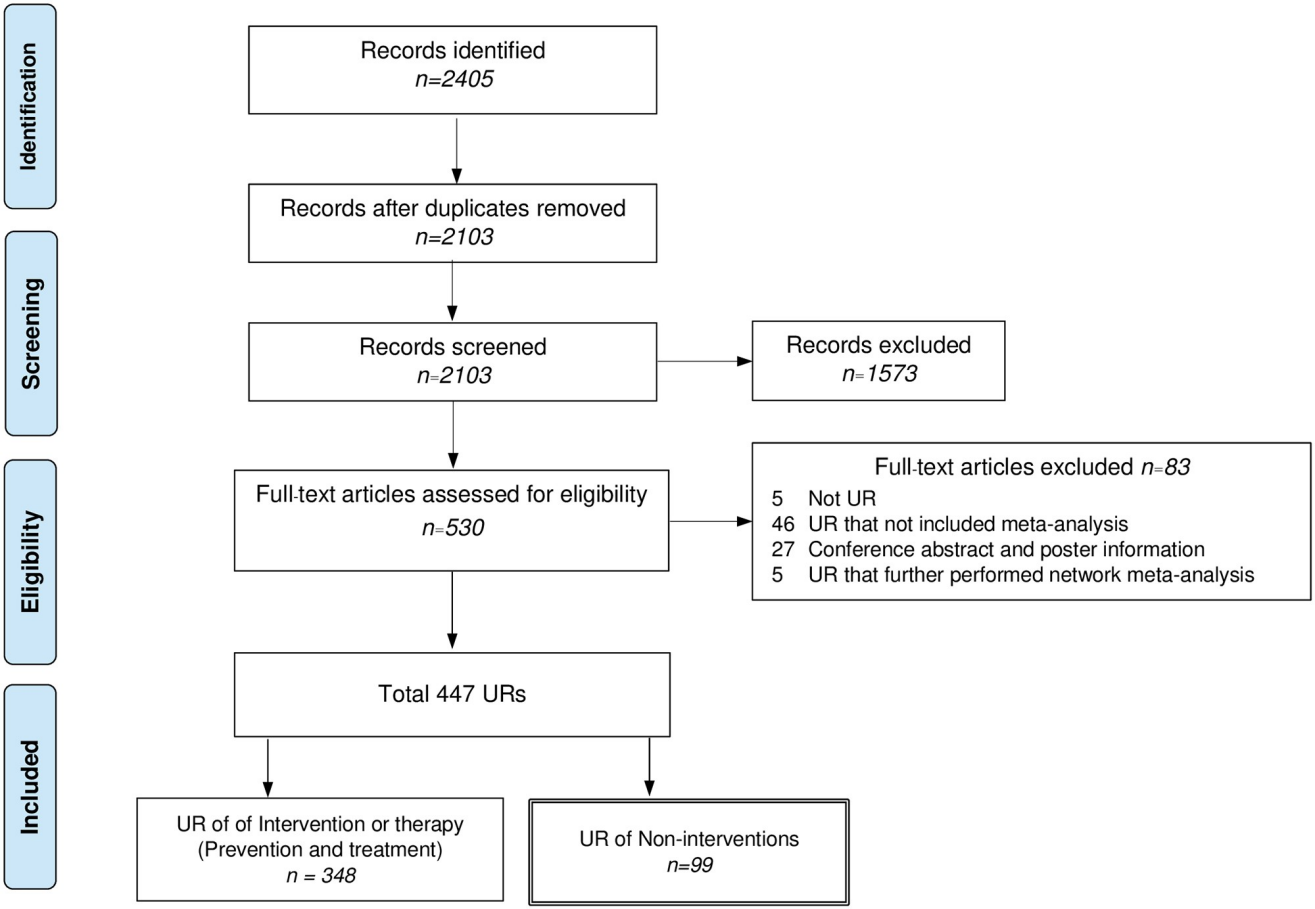
Evidence search and selection.

We identified 2405 articles, of which 302 and 1573 articles were excluded due to duplicates and during screening titles/abstracts, respectively: leaving 530 studies for the full-text review. A total of 447 URs matched with the eligibility criteria. Finally, 348 and 99 URs of intervention and non-therapy/non-intervention studies were eligible but only 99 URs were focused and reported in our scoping review (Fig 1) [13–111]. The reasons for exclusion of the articles after full-text review were described in detail in **S3 Table in** S1 File.

### Characteristics of included URs

Table 1 summarize the characteristics of the 99 included URs. The number of URs increased over time from 1 in 2015 to 32 in 2021, with the majority being published in the recent 5 years (2017–2021), (N = 90, 90.9%).

**Table 1:** Description of included umbrella reviews.

Description	Response	
	N	%
**1. Year published**		
2010–2016	9	9.1
2017–2021	90	90.9
**2. Number of included studies and participants**		
Number of meta-analyses included in URs, median (IQR)	12 (5–42)	
Number of primary studies included in meta-analysis, median (IQR)	243 (174–683)	
Number of study participants, range	8–19,207,552	
Journal impact factor (IF), median (IQR)	4.45 (3.01–7.72)	
**3. Journal publication**		
**3.1 Classified by Top 100 journal ranking**		
Published in High impact groups (Top 100 journal ranking)	7	7.1
Published in lower impact groups	92	92.9
**3.2 Classified by median journal impact factor of included URs**		
Published in High JIF group	48	48.5
Published in lower JIF group	51	51.5
**4. Characteristics of Included meta-analysis**		
URs with meta-analysis of observational studies	78	78.8
URs with meta-analysis of both observational and experimental studies	10	10.1
URs that not reported the study design of primary studies	11	11.1
**5. Certainty of the evidence assessment**		
Assessment was done	56	56.6
**5.1 Tools used for assessing certainty of the evidence (n = 56)**		
Criteria for credibility assessment	45	80.4
GRADE approach	8	14.3
Performed both credibility assessment and GRADE approach	1	1.8
Authors used their own criteria	2	3.6
**6. Methodological quality assessment**		
Assessment was done	74	74.8
**6.1 Tools used for assessing methodological quality**		
AMSTAR	20	27
AMSTAR 2	34	46
JBI critical appraisal checklist for SRs	13	17.6
ROBIS	3	4.1
Other tools	4	5.4
Oxman and Guyatt Overview Quality Assessment Questionnaire (OQAQ)	1	
Authors used their own criteria	1	
A tool developed from the Centre for Reviews and Dissemination (CRD) checklist	1	
Newcastle Ottawa Scale	1	

Most URs (n = 78, 76.8%) included individual SR-MAs of observational studies evaluating risk or protective effects of risk factors, association, and non-modifiable risk factors of a disease or health condition, followed by URs that focused on prevalence/ incidence (n = 11, 11.1%), and etiology, diagnosis/prognostic biomarkers (n = 6, 6.1%). The median number of MAs included in these corresponding URs were 12 (Interquartile range (IQR): 5–42), The median of total number of primary studies included were 243 (IQR: 174–683). The median JIF of URs that included in this study was 4.45 (3.01–7.72). Seven of the 99 URs (7.1%) were published in top-100 ranking journals [30, 31, 41, 71, 95, 100, 103] according to the JIF in 2021.

### Methodological approaches for assessing certainty of the evidence

**Fig 2:**
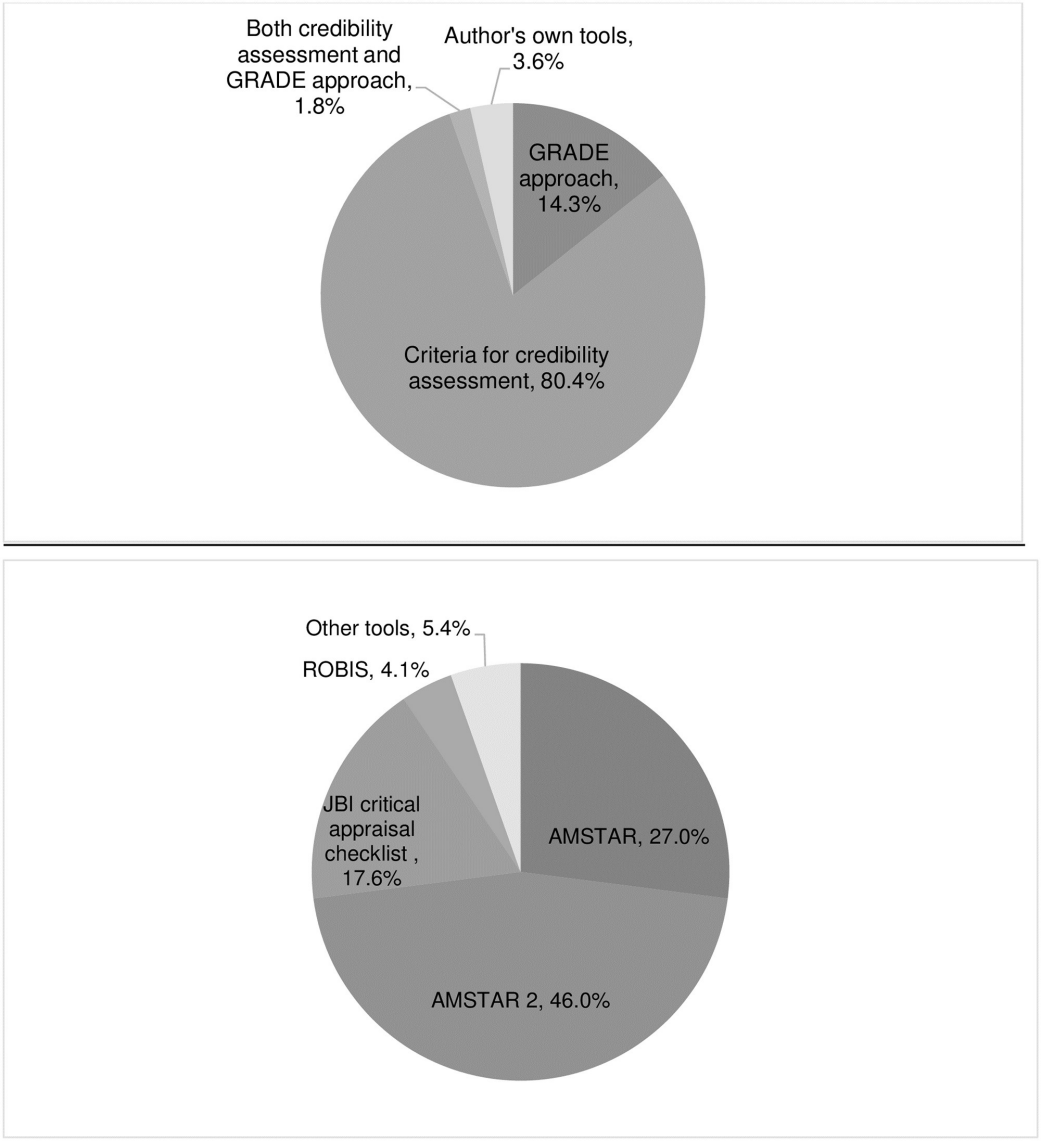
Percent used of methodological approaches for certainty and methodological quality assessment. (A) Methodological approaches for certainty of evidence assessment. (B) Methodological approaches for methodological quality assessment.

Of 99 URs, only half of them (N = 56, 56.6%) assessed the certainty of the evidence, see Table 1, **S4 and S5 Tables in** S1 File. Criteria for credibility assessment was the most frequently used method (N = 45, 80.4%) followed by GRADE approach (N = 8, 14.3%), see Fig 2. Almost all URs used one tool (N = 55, 98.2%), only 1 URs (1.8%) used both criteria for credibility assessment and GRADE approach [61].

**Fig 3:**
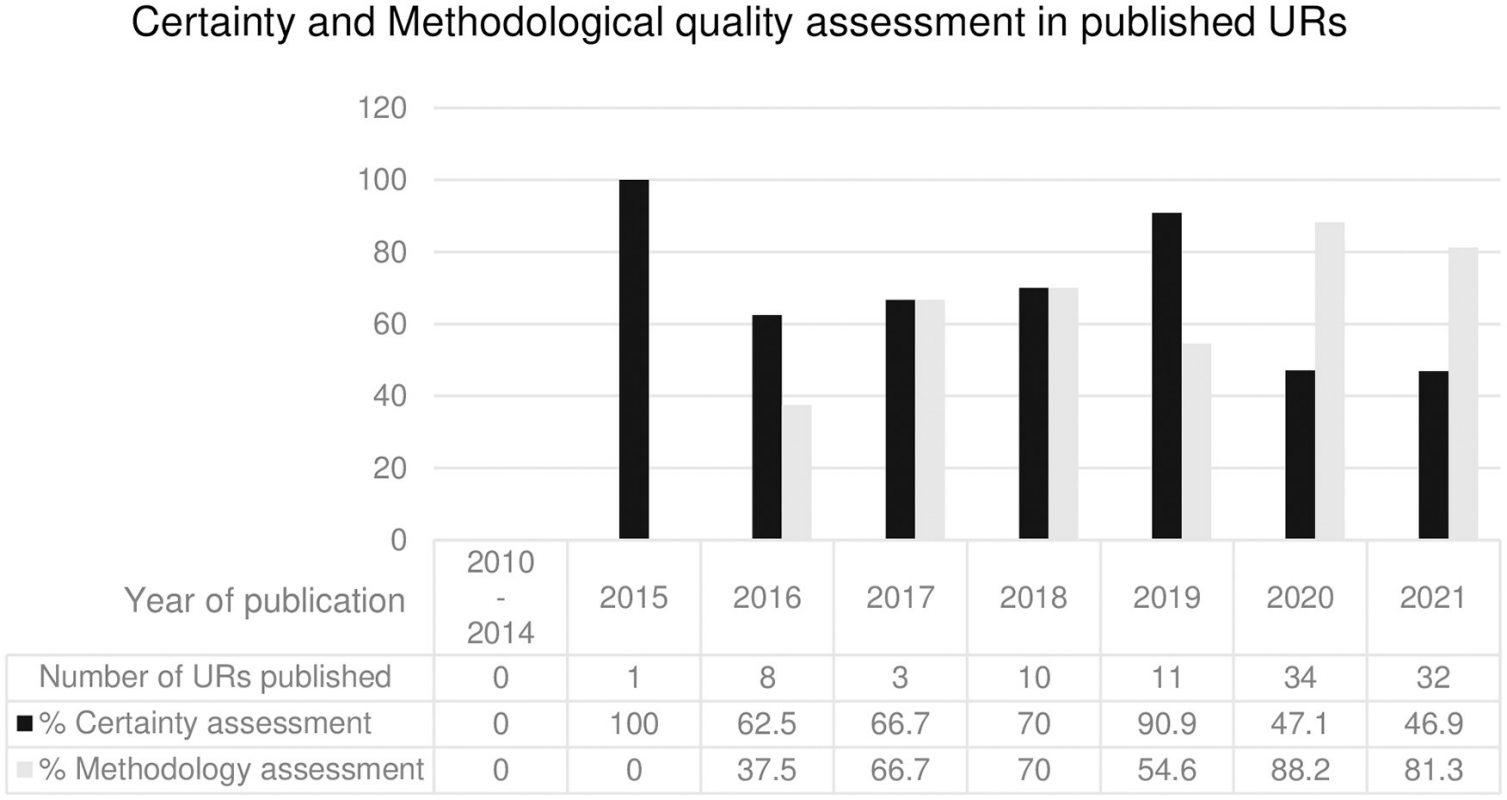
The proportion of studies employing certainty and methodological quality assessment over time.

Table 2 showed the certainty and methodological quality assessment in URs. Based on the median of JIFs, the percent URs that assessed the certainty of evidence was significantly higher in the high impact group (JIF > 4.45) than the lower impact group (JIF ≤ 4.45), i.e., 77.1% vs 37.3%, p < 0.0001. The number of URs that published in top-100 journals group also assessed the certainty of evidence more than the lower impact group, although it was not significant (71.4% vs 55.4%, p = 0.70). Comparing the period of 2010–2016 with 2017–2021, the percent of URs with certainty of the evidence assessments was higher in a period of 2010–2016 in than 2017–2021 but no statistically significant was found (66.7% vs 55.6%, p = 0.73). In addition, Fig 3 showed the direction that the proportion of URs that performed the certainty of evidence assessment was increase over time. Except that only 1 UR that published in 2015 meet our criteria and the author performed certainty of evidence assessment, thus, the proportion of URs that assess the certainty was 100%.

**Table 2:** Certainty and methodological quality assessment in included URs.

Study characteristics	Assessment	P-value
**1. Performed a certainty assessment**		
**1.1 Classified by the median of impact factor**		
Published in Higher impact journals (JIF > 4.45)	37/48 (77.1%)	<0.05^a^
Published in Lower impact journals (JIF ≤ 4.45)	19/51 (37.3%)	
**1.2 Classified by ranking of journal**		
Published in higher impact group (top 100 ranking)	5/7 (71.4%)	0.70^b^
Published in lower impact journals	51/92 (55.4%)	
**1.3 Classified by year of publication**		
2010–2016	6/9 (66.7%)	0.73^b^
2017–2021	50/90 (55.6%)	
**2. Performed a methodological quality assessment**		
**2.1 Classified by the median of impact factor**		
Published in Higher impact journals (JIF > 4.45)	35/48 (72.9%)	0.69^a^
Published in Lower impact journals (JIF ≤ 4.45)	39/51 (74.6%)	
**2.2 Classified by ranking of journal**		
Published in higher impact group (top 100 ranking)	5/7 (71.4%)	0.99^b^
Published in lower impact journals	69/92 (75%)	
**2.3 Classified by year of publication**		
2010–2016	3/9 (33.3%)	<0.05^b^
2017–2021	71/90 (78.9%)	

^a^ Chi-square test,

^b^ Fisher’s exact test.

Of 7 URs published in top-100 ranking journals group [30, 31, 41, 71, 95, 100, 103], the assessment of certainty of the evidence was performed in 5 studies. The most used tools were criteria for credibility assessment (n = 4) followed by 1 study using GRADE approach. However, criteria for credibility assessment used in these URs were varied across studies, as shown in Table 3. For instance, 2 URs used the retained statistical significance in 10% credibility ceiling and the largest study with statistically significant effect as criteria for convincing class (the highest certainty). Four URs classified the certainty of evidence into 5 levels including “Convincing,” Highly suggestive,” Suggestive,” “Weak,” or "Not significant" but 1 UR used this tool to identified associations that had the strongest validity and were not suggestive of bias. Moreover, convincing evidence was graded based on the number of cases included in each MA of ≥ 350 to ≥ 5000 or p-values of < 10^−6^ or even < 0.001, as shown in **S6 Table in** S1 File.

**Table 3:** Details of the criteria of credibility assessment used in umbrella reviews published in the top-100 ranking journals*.

Details of criteria	Criteria for credibility assessment of each study			
	Belbasis L, 2015 [71]	Radua J, 2018 [103]	Kim JY, 2019 [95]	Kim JH, 2020 [30]
1. Number of categories (Label used)	**2 Identified associations that had the strongest validity and were not suggestive of bias**	5 (Convincing, Highly suggestive, Suggestive, Weak, Not significant)	5 (Convincing, Highly suggestive, Suggestive, Weak, Not significant)	5 (Convincing, Highly suggestive, Suggestive, Weak, Not significant)
**2. Details of each category**				
**2.1 Convincing/class I**				
**• Number of cases**	> 1000	> 1000	> 1000	> 1000
**• P-value**	fixed-effects and random-effects at p<0.05 and at p<0.001	P < 10^−6^	P < 10^−6^	Random effects P < 10^−6^
**• 95% prediction interval excluded null**	Used	Used	Used	Used
**• Heterogeneity**	I^2^ < 50%	I^2^ < 50%	I^2^ < 50%	I^2^ < 50%
**• No evidence of small-study effects**	Used	Used	Used	Used
**• No evidence of excess significance bias**	Not Used	Not Used	**Used**	**Used**
**• Retained statistical significance in 10% credibility ceiling**	Not Used	Not Used	**Used**	**Used**
**• Largest study with statistically significant effect**	Not Used	Not Used	**Used**	**Used**
**2.2 Highly suggestive/class II**				
**• Number of cases**	No category	> 1000	> 1000	> 1000
**• P-value**	No category	P < 10^−6^	P < 10^−6^	Random effects P < 10^−6^
**• Largest study with statistically significant effect**	No category	Used	Used	Used
**2.3 Suggestive/class III**				
**• Number of cases**	No category	> 1000	> 1000	> 1000
**• P-value**	No category	P < 10^−3^	P < 10^−3^	P < 10^−3^
**2.4 Weak/class IV**				
**• P-value**	No category	P ≤ 0.05	P ≤ 0.05	P ≤ 0.05
**2.5 Non-significant**				
**P-value**	No category	P > 0.05	P > 0.05	P > 0.05

* Of 7 umbrella reviews published Top-100 ranking journals group that included in this study, 4 studies used the criteria of credibility assessment to assess the certainty of the evidence.

### Methodological quality assessment

Most of the included URs performed the methodological quality assessment of included MAs (n = 74, 74.8%). Of these, the most frequently used tool was AMSTAR 2 (n = 34, 46%), followed by its old version called AMSTAR (n = 20, 27%), and Joanna Bring Institute (JBI) critical appraisal checklist for SRs (n = 13, 17.6%), as shown in Table 1 and Fig 2.

Among 7 URs that published in top-100 journal ranking group [30, 31, 41, 71, 95, 100, 103], 5 of them (71.4%) assessed the methodological quality using AMSTAR [100, 103] and AMSTAR 2 [30, 41, 95]. The proportion of URs in the high-impact journal group, based on the median of journal impact factor, that performed the methodological quality assessment was lower than those published in the lower impact journal group, but it was not statistically significant (72.9% vs 74.6%, p = 0.69), as shown in Table 3. The more recent URs published in 2017–2021, performed a methodological quality assessment more often than those published in 2010–2016 significantly, (78.9% vs 33.3%, p < 0.05). Furthermore, Fig 3 also showed the inclination of the proportion of URs that performed the methodological assessment over time.

## Discussion

To the best of our knowledge, this is the first study identifying the methodological approaches for assessing the certainty of evidence in URs of SR-MAs considering 99 URs of non-intervention studies. Our finding suggested that only nearly half of the included URs assessed certainty of evidence, in which the criteria of credibility assessment was a mainly used tool. URs that were published in high JIFs and high-ranking journals are more likely to assess the certainty of evidence than URs published in lower JIFs and lower-ranking journals. Nearly 80% of the URs performed a methodological quality assessment and the AMSTAR 2 was the most frequently used tool for this process.

URs were increasingly published over the last decade to compile evidence and provide broad pictures of information from SR-MAs [2]. URs of epidemiological investigations and non-interventional studies also help in establish evidence linking exposure to the incidence of certain health condition in a population. Consequently, these studies are expected to play a key role in gauging the burden of diseases, understand the risk or protective factors, delineating guidelines for prevention as well as streamlining the treatment development process [3, 4]. The certainty of the evidence from these URs, which is the extent of confidence to support a decision or recommendations, may further be used as supportive evidence in develop clinical practice guidelines and recommendations. High certainty in evidence means that the investigators are very confident that the effect they found across studies is close to the true effect and vice versa [111]. URs of these studies should aim to provide the highest certainty of evidence to facilitate better health outcomes. Despite the necessity of assessing the certainty of the evidence in URs, there is no consensus that which approach should be the method of choice. Although Aromataris et al.—a methodology working group formed by the JBI (formerly named the URs Working Group)—published the guidance on how to conduct and report an UR [6], the methodology for the certainty assessment was not provided. Hence, we included URs of non-interventional studies in this scoping review to provide information the methodology for the certainty assessment that used these days.

According to our findings, approximately half of the included URs assessed the certainty of evidence, with the criteria of credibility assessment being the most commonly utilized tool. In contrast to the results from a previous study by Hartling et al [1] indicating that described the methodological approaches in overviews of interventions. They indicated that only 16% of the overview of reviews published between 2000 and 2011 assessed the certainty of the evidence and the most frequently used method is the GRADE approach. One of the reasons could be that the GRADE approach is a well-established tool developed to determine the certainty of evidence-based on several factors namely risk of bias, imprecision, indirectness, inconsistency, and publication bias [111]. However, GRADE approach was primarily designed for assessing the quality of the evidence from primary studies. Further guidance is needed to ensure appropriate use and interpretation of the GRADE tool when it is applied to assess the quality of evidence of SRs, instead of primary studies [1]. The other reasons that our study differs from the previous study likely because we specifically considered the URs that included MAs of non-interventions. The criteria for credibility assessment classified the certainty of the evidence according to several statistical criteria, which usually reported in MAs. This method was recently released [6, 112], might be specific to URs of MAs of non-interventions, and was being used more commonly.

Because most researchers generally aspire to publish their research findings in the top journal publishers. We then classified URs that included in our study into high and lower impact groups using journal impact factors (JIFs) to find the impact of methodological approaches on journal publication. The JIFs help reflect several factors such as the high frequency of citations, media promotion of articles and journals, and the increase in speed of the review and publication process [113, 114]. Although JIFs could not be used to reflect the full impact of journals on formal implications, it remains an acceptable objective and quantifiable measure of knowledge dissemination nowadays [113–115]. This study demonstrated that a higher number of URs with a certainty assessment was published in higher impact and higher-ranking journals. One of the reasons could be that the assessment helped to reflect the certainty of results and facilitate the translation of the evidence into guideline recommendations.

When focusing in the URs that published in top-100 ranking journal, criteria for credibility assessment was also the most used method. However, levels of evidence and a series of statistical tests in these URs using the arbitrary cut-off values and the cut point of each component in these criteria varied slightly. As shown in Table 3, UR that published earlier start using criteria for credibility assessment to identify associations that had the strongest certainty or not. Then, the URs published later used criteria for credibility assessment to categorize the certainty of evidence into several levels from convincing (the highest level) to weak (the lowest level). Their classification was obtained through strict criteria including number of cases and several statistical parameters. The degree of statistical significance (p-value) was used despite the lack of consensus on what might be and optimal threshold and p-value thresholds might need to be tailored to the specific research setting and even to a specific database [2, 116]. Some URs used p<0.05 or p<0.001, but several URs used the stricter level of P < 10^−6^ to be categorized as the highest certainty of evidence. Several other parameters also have been used including a heterogeneity, predictive interval, and small-study effect test. The excess significance bias, which evaluates whether the number of observed studies with statistically significant results differs from the expected number of positive studies [117], has also been used to be a criterion in some of the URs. The credibility ceiling is a method to test whether the observational studies can survive the specific level that the likelihood of summary effect being in a specific direction [118]. Two URs that published more recently used the retained statistical significance in 10% credibility ceiling as criteria for convincing class (the highest certainty) likely because this method was published more recently [118, 119]. Moreover, criteria of credibility assessment were generally reproduced following previous URs, where some of the published URs were repeatedly cited [71, 79, 80]. Therefore, our findings highlighted the importance of guidance for assessing the certainty of the evidence in URs to recommend the most appropriate tools to provide standards for those conducting URs.

This study also demonstrated that majority of the included URs performed a methodological quality assessment. This was more frequent than a previous study [1] that reported the assessment of methodological quality in only 37% of the overviews of reviews. One of the reasons could be that this process has been strongly recommended in the methodological guidance for producing URs [2] and has been implemented longer than the certainty assessment. This process is essential to ensure that the methodological quality of SR-MAs that included in URs are adequately assessed and incorporated into the results and conclusions. Besides, we found that the most often used tool for methodological quality assessment changed from the Oxman and Guyatt Overview Quality Assessment Questionnaire (OQAQ) to AMSTAR. The AMSTAR tool has been recommended since 2007 and the revised version-AMSTAR 2 was released in 2016 [12]. Given that the revised tool introduced recently, the method advocated in published guidance have evolved over time and the variation of tool used for methodological quality assessment reported in this study confirms the need for updated guidance for conducting URs. In addition, our findings also highlighted that many of the published URs of non-intervention studies performed the certainty of evidence and/or methodological quality assessments, particularly in the more recent published URs and URs that published in journals with higher impact factor. In the current time, the criteria for credibility assessments is the most commonly used methods for certainty assessment and AMSTAR-2 is the most used methods for methodological quality assessments. These results help emphasize the future researchers to apply these assessments in their studies.

Our study has some limitations. First, the definition of included studies was restricted to URs. This might not cover all types of other kinds of reviews for example- overview of reviews, and review of reviews. Therefore, our findings with regards to terminology used to describe “umbrella reviews” and methods used might not be comprehensive or wholly representative. However, there is no universally accepted technical term for this new type of reviews that summarize or synthesize findings from systematic reviews. The term URs has been used increasingly and studies that describe the methodological approach regarding the URs are sparse to date. Second, this study is confined to only URs of non-intervention studies. The methods used for assessing the certainty of evidence and methodological quality in URs that contained other study designs might be different from our findings and could be extended in future research. Third, our study focused on describing the method used in previously published URs and most of them did not provide the reasons for methods selection. Thus, we could not assess the reasons why each UR used different approaches for assessing the certainty of evidence and methodological quality. Although this study could not endorse which method is the best for the certainty of the evidence assessment in URs, a major strength of our study is that it provides a broad picture of the certainty assessment methods used in URs and the commonly used tools to perform this assessment.

## Conclusions

This study revealed that only half of URs that included MAs of non-interventional studies have assessed the certainty of the evidence. The criteria of credibility assessment were the most used method. Moreover, URs that published in higher impact journals assessed the certainty of evidence more than the lower impact group. Therefore, guidance and standards are required to ensure the methodological rigor and consistency of certainty of evidence assessment for URs.

## Supporting information

S1 File (DOCX)

## Abbreviations

AMSTAR: A MeaSurement Tool to Assess systematic Reviews
GRADE: Grading of Recommendations, Assessment, Development and Evaluations
IQR: Interquartile range
JBI: Joanna Bring Institute
JIF: journal impact factors
MA: meta-analysis
OQAQ: Oxman and Guyatt Overview Quality Assessment Questionnaire
RCT: randomized controlled trials
ROBIS: A Risk of Bias Assessment Tool for Systematic Reviews
SR: systematic reviews
UR: umbrella review
