# Supplementary material for: Methodological approaches for assessing certainty of the evidence in umbrella reviews: A scoping review
Source: PLoS One. 2022 Jun 8;17(6):e0269009. doi: 10.1371/journal.pone.0269009 (PMC9176806; doi:10.1371/journal.pone.0269009)
Supplement: S1 File — (DOCX) [file pone.0269009.s001.docx]

**Supplementary file**

**Methodological approaches for assessing certainty of the evidence in umbrella reviews: A scoping review**

Saranrat Sadoyu, Kaniz Afroz Tanni, Nontaporn Punrum, Sobhon Paengtrai, Warittakorn Kategaew, Nattiwat Promchit, Nai Ming Lai, Ammarin Thakkinstian, Surachat Ngorsuraches, Mukdarut Bangpan, Sajesh Veettil, Nathorn Chaiyakunapruk

**Supplementary content**

**Contents Page no.**

**Tables**

**S1 Table** Preferred Reporting Items for Systematic reviews and Meta-Analyses extension for Scoping Reviews (PRISMA-ScR) Checklist 1

**S2 Table** Search Strategy 4

## **S3 Table** Studies excluded after full-text revision, with reasons for exclusion 5-8

**S4 Table** Characteristics of included umbrella review 9-16

## **S5 Table** Details of method used for certainty and methodological quality 17-20

## assessment in included umbrella reviews

## **S6 Table** Details of criteria for credibility assessment used in included URs 21-22

**Files**

## **S1 file** Details of data extraction 5

## **S2 file** Protocol 23-27

**S1 Table** Preferred Reporting Items for Systematic reviews and Meta-Analyses extension for Scoping Reviews (PRISMA-ScR) Checklist

| **SECTION** | **ITEM** | **PRISMA-ScR CHECKLIST ITEM** | **REPORTED ON PAGE #** |
| --- | --- | --- | --- |
| **TITLE** | | | |
| Title | 1 | Identify the report as a scoping review. | 1 |
| **ABSTRACT** | | | |
| Structured summary | 2 | Provide a structured summary that includes (as applicable): background, objectives, eligibility criteria, sources of evidence, charting methods, results, and conclusions that relate to the review questions and objectives. | 2 |
| **INTRODUCTION** | | | |
| Rationale | 3 | Describe the rationale for the review in the context of what is already known. Explain why the review questions/objectives lend themselves to a scoping review approach. | 4-5 |
| Objectives | 4 | Provide an explicit statement of the questions and objectives being addressed with reference to their key elements (e.g., population or participants, concepts, and context) or other relevant key elements used to conceptualize the review questions and/or objectives. | 5 |
| **METHODS** | | | |
| Protocol and registration | 5 | Indicate whether a review protocol exists; state if and where it can be accessed (e.g., a Web address); and if available, provide registration information, including the registration number. | 5 |
| Eligibility criteria | 6 | Specify characteristics of the sources of evidence used as eligibility criteria (e.g., years considered, language, and publication status), and provide a rationale. | 6 |
| Information sources* | 7 | Describe all information sources in the search (e.g., databases with dates of coverage and contact with authors to identify additional sources), as well as the date the most recent search was executed. | 5-6 |
| Search | 8 | Present the full electronic search strategy for at least 1 database, including any limits used, such that it could be repeated. | S2 Table |
| Selection of sources of evidence† | 9 | State the process for selecting sources of evidence (i.e., screening and eligibility) included in the scoping review. | 6 |
| Data charting process‡ | 10 | Describe the methods of charting data from the included sources of evidence (e.g., calibrated forms or forms that have been tested by the team before their use, and whether data charting was done independently or in duplicate) and any processes for obtaining and confirming data from investigators. | 7 |
| Data items | 11 | List and define all variables for which data were sought and any assumptions and simplifications made. | 6-7 |
| Critical appraisal of individual sources of evidence§ | 12 | If done, provide a rationale for conducting a critical appraisal of included sources of evidence; describe the methods used and how this information was used in any data synthesis (if appropriate). | N/A |
| Synthesis of results | 13 | Describe the methods of handling and summarizing the data that were charted. | 7 |
| **RESULTS** | | | |
| Selection of sources of evidence | 14 | Give numbers of sources of evidence screened, assessed for eligibility, and included in the review, with reasons for exclusions at each stage, ideally using a flow diagram. | 8, Figure 1, S3 Table |
| Characteristics of sources of evidence | 15 | For each source of evidence, present characteristics for which data were charted and provide the citations. | 8 |
| Critical appraisal within sources of evidence | 16 | If done, present data on critical appraisal of included sources of evidence (see item 12). | N/A |
| Results of individual sources of evidence | 17 | For each included source of evidence, present the relevant data that were charted that relate to the review questions and objectives. | 10-14 |
| Synthesis of results | 18 | Summarize and/or present the charting results as they relate to the review questions and objectives. | 11 |
| **DISCUSSION** | | | |
| Summary of evidence | 19 | Summarize the main results (including an overview of concepts, themes, and types of evidence available), link to the review questions and objectives, and consider the relevance to key groups. | 14 |
| Limitations | 20 | Discuss the limitations of the scoping review process. | 18-19 |
| Conclusions | 21 | Provide a general interpretation of the results with respect to the review questions and objectives, as well as potential implications and/or next steps. | 19 |
| **FUNDING** | | | |
| Funding | 22 | Describe sources of funding for the included sources of evidence, as well as sources of funding for the scoping review. Describe the role of the funders of the scoping review. | 19 |

JBI = Joanna Briggs Institute; PRISMA-ScR = Preferred Reporting Items for Systematic reviews and Meta-Analyses extension for Scoping Reviews.

* Where *sources of evidence* (see second footnote) are compiled from, such as bibliographic databases, social media platforms, and Web sites.

† A more inclusive/heterogeneous term used to account for the different types of evidence or data sources (e.g., quantitative and/or qualitative research, expert opinion, and policy documents) that may be eligible in a scoping review as opposed to only studies. This is not to be confused with *information sources* (see first footnote).

‡ The frameworks by Arksey and O’Malley (6) and Levac and colleagues (7) and the JBI guidance (4, 5) refer to the process of data extraction in a scoping review as data charting*.*

§ The process of systematically examining research evidence to assess its validity, results, and relevance before using it to inform a decision. This term is used for items 12 and 19 instead of "risk of bias" (which is more applicable to systematic reviews of interventions) to include and acknowledge the various sources of evidence that may be used in a scoping review (e.g., quantitative and/or qualitative research, expert opinion, and policy document).

*From:* Tricco AC, Lillie E, Zarin W, O'Brien KK, Colquhoun H, Levac D, et al. PRISMA Extension for Scoping Reviews (PRISMAScR): Checklist and Explanation. Ann Intern Med. 2018;169:467–473. [doi: 10.7326/M18-0850](http://annals.org/aim/fullarticle/2700389/prisma-extension-scoping-reviews-prisma-scr-checklist-explanation).

## **S2 Table** Search Strategy

| **Databases** | **Search strategy** |
| --- | --- |
| Embase via Ovid | ('umbrella review*':ti,ab,kw OR ('umbrella*':ti,ab AND 'review*':ti,ab)) AND [embase]/lim NOT [medline]/lim AND [1-5-2010]/sd NOT [30-9-2021]/sd |
| PubMed | (("umbrella review"[Title/Abstract] OR "umbrella"[Title/Abstract]) AND (("review"[Title/Abstract] OR "review literature as topic"[MeSH Terms]) OR "review"[Publication Type])) AND (("2010/05/01"[Date - Publication] : "2021/09/30"[Date - Publication])) |
| The Cochrane Library | umbrella review, filter publication date 1/5/2010-30/9/2021 |

## **S1 file** Details of data extraction

The following data were extracted: first author, year of publication, study design, number of included SR-MAs, the study design of included studies in the SR-MAs (e.g., RCTs, non-RCTs, mixed RCTs and non-RCTs), characteristics of PICO (participants, interventions/exposures, comparators, and outcomes), method/approach/tool used for assessing the certainty of the evidence of the URs (e.g., GRADE approach, and criteria for credibility assessment) and the methods used to assess the methodological quality of the included SRs/MAs (e.g., A Measurement Tool to Assess systematic Reviews (AMSTAR), Oxman and Guyatt Overview Quality Assessment Questionnaire (OQAQ), etc.).

The assessment of the certainty of evidence was defined as any of evaluation of the totality or strength of the evidence such as the GRADE approach, criteria for credibility assessment, Agency for Healthcare Research and Quality (AHRQ) methods for systematic review, and other approaches used to grade the overall body of the UR evidence.

## **S3 Table** Studies excluded after full-text revision, with reasons for exclusion

| **First Author** | **Year published** | **Title** |
| --- | --- | --- |
| **1.Not the URs that included meta-analyses** | | |
| Javed S | 2020 | Efficacy and safety profile of proteasome inhibitor based drug regimens for treatment of newly diagnosed AL amyloidosis: A systematic review |
| Owoeye OBA | 2020 | Reducing Injuries in Soccer (Football): an Umbrella Review of Best Evidence Across the Epidemiological Framework for Prevention |
| Beshir SA | 2021 | Umbrella Review on Non-Statin Lipid-Lowering Therapy |
| Janiaud P | 2021 | Validity of observational evidence on putative risk and protective factors: appraisal of 3744 meta-analyses on 57 topics |
| Subrata SA | 2021 | The nursing outlook of the self- and family management support programs among Indonesian with diabetes: An umbrella review |
| Gartlehner G | 2010 | Differences in efficacy and safety of pharmaceutical treatments between men and women: an umbrella review |
| Safron M | 2011 | Micro-environmental characteristics related to body weight, diet, and physical activity of children and adolescents: a systematic umbrella review |
| Mbemba G | 2013 | Interventions for supporting nurse retention in rural and remote areas: an umbrella review |
| Murad MH | 2014 | Using GRADE for evaluating the quality of evidence in hyperbaric oxygen therapy clarifies evidence limitations |
| Cairns J | 2015 | Go slow: an umbrella review of the effects of 20 mph zones and limits on health and health inequalities |
| Gil-GonzÃ¡lez D | 2015 | Is health a right for all? An umbrella review of the barriers to health care access faced by migrants |
| Hasan R | 2016 | A systematic review and meta-analysis of glycemic control for the prevention of diabetic foot syndrome |
| Househ M | 2016 | The role of short messaging service in supporting the delivery of healthcare: An umbrella systematic review |
| Wiechula R | 2016 | Umbrella review of the evidence: what factors influence the caring relationship between a nurse and patient? |
| Winters S | 2016 | Cross-sector Service Provision in Health and Social Care: An Umbrella Review |
| Corso E | 2017 | Enhanced recovery after elective caesarean: a rapid review of clinical protocols, and an umbrella review of systematic reviews |
| Olry de Labry Lima A | 2017 | [Identification of health outcome indicators in Primary Care. A review of systematic reviews] |
| Salleh S | 2017 | Simulation Modelling in Healthcare: An Umbrella Review of Systematic Literature Reviews |
| Shepherd-Banigan M | 2017 | Improving vasomotor symptoms; psychological symptoms; and health-related quality of life in peri- or post-menopausal women through yoga: An umbrella systematic review and meta-analysis |
| Thomson, K. | 2017 | The effects of public health policies on health inequalities: A review of reviews |
| Bird EL | 2018 | Built and natural environment planning principles for promoting health: an umbrella review |
| Buja A | 2018 | Developing a new clinical governance framework for chronic diseases in primary care: an umbrella review |
| De Sio S | 2018 | Ergonomic risk and preventive measures of musculoskeletal disorders in the dentistry environment: an umbrella review |
| O'Donoghue G | 2018 | Socio-economic determinants of physical activity across the life course: A "DEterminants of DIet and Physical ACtivity" (DEDIPAC) umbrella literature review |
| Puggina A | 2018 | Policy determinants of physical activity across the life course: a 'DEDIPAC' umbrella systematic literature review |
| Thomson K | 2018 | The effects of public health policies on health inequalities in high-income countries: an umbrella review |
| Woldie M | 2018 | Community health volunteers could help improve access to and use of essential health services by communities in LMICs: an umbrella review |
| Ying J | 2018 | Iterating a framework for the prevention of caregiver depression in dementia: a multi-method approach |
| Ãlvarez-Gallardo IC | 2019 | Therapeutic validity of exercise interventions in the management of fibromyalgia |
| Barnett MJ | 2019 | Barriers and enablers for smoking cessation amongst pregnant women: An Umbrella Review |
| Duan-Porter W | 2019 | Systematic Review: Risk Factors and Interventions to Prevent or Delay Long-term Nursing Home Placement for Adults with Impairments |
| Duley L | 2019 | Improving quality of care and outcome at very preterm birth: the Preterm Birth research programme, including the Cord pilot RCT |
| Krishnan JA | 2019 | An Umbrella Review: corticosteroid Therapy for Adults with Acute Asthma |
| McMahon N | 2019 | Effects of prevention and harm reduction interventions on gambling behaviours and gambling related harm: An umbrella review |
| Naik Y | 2019 | Going upstream - an umbrella review of the macroeconomic determinants of health and health inequalities |
| Ramalho A | 2019 | Primary health care quality indicators: An umbrella review |
| Thompson W | 2019 | Factors associated with antibiotic prescribing for adults with acute conditions: an umbrella review across primary care and a systematic review focusing on primary dental care |
| Alsulamy N | 2020 | What Influences the Implementation of Shared Decision Making: An Umbrella Review |
| Bressan V | 2020 | Nursing handovers and patient safety: Findings from an umbrella review |
| Carini E | 2020 | Assessing hospital performance indicators. What dimensions? Evidence from an umbrella review |
| De Sio S | 2020 | Work-related stress risk and preventive measures of mental disorders in the medical environment: an umbrella review |
| Fennelly O | 2020 | Successfully implementing a national electronic health record: a rapid umbrella review |
| Malik RF | 2020 | What do we really assess with organisational culture tools in healthcare? An interpretive systematic umbrella review of tools in healthcare |
| Santaniello A | 2020 | Methodological and Terminological Issues in Animal-Assisted Interventions: An Umbrella Review of Systematic Reviews |
| Wendrich-van Dael A | 2020 | Advance care planning for people living with dementia: An umbrella review of effectiveness and experiences |
| Einav S | 2021 | Vasopressor and inotrope treatment for septic shock: An umbrella review of reviews |
| Ghassab-Abdollahi N | 2021 | The effects of Huperzine A on dementia and mild cognitive impairment: An overview of systematic reviews |
| Park EJ | 2021 | Advance care planning for older adults in community-based settings: An umbrella review |
| Pinilla A | 2021 | Experimental and computational advances on the study of Viscous Fingering: An umbrella review |
| SmithBattle L | 2021 | An Umbrella Review of Qualitative Research on Teen Mothering |
| Zisis E | 2021 | Climate change, 24-hour movement behaviors, and health: a mini umbrella review |
| **2. Materials for conference/ poster presentations** | | |
| Campbell, J. | 2016 | The pharmacogenomics of chemotherapy-induced toxicities: An umbrella review of systematic reviews and meta-analyses |
| Kalliala, I. | 2016 | Obesity and gynaecological cancer-an umbrella review and meta-analysis |
| Alexander, L. | 2017 | Effectiveness of vocational rehabilitation on work participation in adults with musculoskeletal disorders: An umbrella review |
| Giannakou, K. | 2017 | Non-genetic risk factors for gestational diabetes: An umbrella review of systematic reviews and meta-analyses of observational studies |
| Kim, S. | 2017 | An umbrella review of systematic reviews of pharmacological treatments post-TBI |
| Tobon-Trujillo, M. | 2017 | Direct oral anticoagulants for the treatment of venous thromboembolism: An umbrella review of meta-analysis and economic studies |
| Alhasani | 2018 | Mobility measures among individuals with acquired brain injury (ABI): An umbrella review |
| Castricum, A. | 2018 | Exercise as medicine – Evidence for prescribing exercise for Australia's nine national health priority areas: An umbrella review |
| Martínez-Rodríguez, R. | 2018 | Association between the consumption of sugar-sweetened beverages and hypertension: Umbrella review |
| Toonmala, K. | 2018 | Effects of Turmeric on Clinical Conditions: Umbrella Reviews of Systematic Review and Meta-Analysis |
| Ambler, G. K. | 2019 | Antiplatelet Therapy in Peripheral Arterial Disease: An Umbrella Review and Meta-analysis of Preventative and Treatment Outcomes |
| Anstey, K. J. | 2019 | Limited global representativeness of evidence on risk factors for dementia: a challenge for developing risk reduction guidelines |
| Bardou, M. | 2019 | Chemoprevention of colorectal cancer in the general population: an umbrella metaanalysis |
| Dombrovskiy, V. | 2019 | Efficacy and safety of mechanical thrombectomy in patients with ischemic stroke: an umbrella systematic review |
| Jebara, T. | 2019 | Exploring perspectives around non-medical prescribing: An umbrella review |
| Keane, M. | 2019 | Integrated interventions to reduce pressure on acute hospitals: A systematic umbrella review |
| Semertzidou, A | 2019 | Risk factors for ovarian cancer: An umbrella review |
| Semertzidou, A. | 2019 | Diabetes and gynaecological cancers: An umbrella review |
| Valenzuela PL | 2019 | Effectiveness of multicomponent lower extremity injury prevention programmes in team-sport athletes: an umbrella review |
| Dinu M | 2020 | Effects of popular diets on anthropometric and metabolic parameters: An umbrella review of meta-analyses of randomized controlled trials |
| Freitag A | 2020 | PNS156 Are Surrogate Outcomes Enough for the Reimbursement of Health Technologies? An Umbrella Literature Review |
| Kemp E | 2020 | Interventions to manage cardiovascular disease risk in cancer patients and survivors: An umbrella review |
| Valenzuela PL | 2020 | Infographic. Effectiveness of multicomponent lower extremity injury prevention programmes in team-sport athletes: an umbrella review |
| Andrade A | 2021 | Infographic. Effects of exercise in patients with fibromyalgia: an umbrella review |
| Jayedi A | 2021 | Individual nutrients, foods, and dietary programs for weight loss in overweight and obese adults: An umbrella review of 76 meta-analyses reporting 92 dietary interventions among 43,870 participants |
| Michels N | 2021 | Microbiome and metabolic health: An umbrella review of observational and interventional studies |
| Shao S | 2021 | Sglt2 inhibitors in type 2 diabetes patients with non-alcoholic fatty liver diseases: An umbrella review of systematic reviews |
| **3. URs that further performed network meta-analysis** | | |
| Goldstein KM | 2017 | Use of mindfulness, meditation and relaxation to treat vasomotor symptoms |
| Biondi-Zoccai G | 2018 | Umbrella review and multivariate meta-analysis of diagnostic test accuracy studies on hybrid non-invasive imaging for coronary artery disease |
| Correll CU | 2021 | Efficacy and acceptability of pharmacological, psychosocial, and brain stimulation interventions in children and adolescents with mental disorders: an umbrella review |
| Suso-Martí L | 2021 | Effectiveness of Telerehabilitation in Physical Therapist Practice: An Umbrella and Mapping Review With Meta-Meta-Analysis |

## **S4 Table** Characteristics of included umbrella reviews

| **First author** | **Publication year** | **Journal published** | **Title of study** | **No.of included MAs (median)** | **No. of primary studies included in MAs (median)** |
| --- | --- | --- | --- | --- | --- |
| Belbasis L | 2015 | Lancet Neurology | Environmental risk factors and multiple sclerosis: an umbrella review of systematic reviews and meta-analyses | 17 | 416 |
| Apostolo J | 2016 | JBI Database of Systematic Reviews and Implementation Reports | Predicting risk and outcomes for frail older adults: an umbrella review of frailty screening tools | 5 | NR |
| Belbasis L | 2016 | Neuro epidemiology | Environmental Risk Factors and Amyotrophic Lateral Sclerosis: An Umbrella Review and Critical Assessment of Current Evidence from Systematic Reviews and Meta-Analyses of Observational Studies | 85 | 138 |
| Belbasis L | 2016 | J Dermatol Sci | Non-genetic risk factors for cutaneous melanoma and keratinocyte skin cancers: An umbrella review of meta-analyses | 10 | NR |
| Belbasis L | 2016 | BMC Med | Birth weight in relation to health and disease in later life: an umbrella review of systematic reviews and meta-analyses | 78 | NR |
| Bellou V | 2016 | Parkinsonism Relat Disord | Environmental risk factors and Parkinson's disease: An umbrella review of meta-analyses | 32 | NR |
| Campbell JM | 2016 | Cancer Chemother Pharmacol | Methotrexate-induced toxicity pharmacogenetics: an umbrella review of systematic reviews and meta-analyses | 19 | NR |
| Campbell JM | 2016 | Pharmacogenomics J | Fluoropyrimidine and platinum toxicity pharmacogenetics: an umbrella review of systematic reviews and meta-analyses | 6 | NR |
| Carvalho AF | 2016 | Psychol Med | Bias in emerging biomarkers for bipolar disorder | 20 | NR |
| Bellou V | 2017 | Alzheimer’s & Dementia | Systematic evaluation of the associations between environmental risk factors and dementia: An umbrella review of systematic reviews and meta-analyses | 76 | 691 |
| Bortolato B | 2017 | Bipolar Disord | Systematic assessment of environmental risk factors for bipolar disorder: an umbrella review of systematic reviews and meta-analyses | 7 | 54 |
| Campbell JM | 2017 | Pharmacogenomics J | Irinotecan-induced toxicity pharmacogenetics: an umbrella review of systematic reviews and meta-analyses | 17 | NR |
| Belbasis L | 2018 | Acta Psychiatr Scand | Risk factors and peripheral biomarkers for schizophrenia spectrum disorders: an umbrella review of meta-analyses | 98 | NR |
| Belbasis L | 2018 | Int J Rheum Dis | Elucidating the environmental risk factors for rheumatic diseases: An umbrella review of meta-analyses | 42 | 216 |
| Bellou V | 2018 | PLOS One | Risk factors for type 2 diabetes mellitus: An exposure-wide umbrella review of meta-analyses | 142 | NR |
| Cupp, M. | 2018 | British Journal of Cancer | Neutrophil counts and cancer prognosis: An umbrella review of systematic reviews and meta-analyses of observational studies | 81 | NR |
| Giannakou K | 2018 | Ultrasound Obstet Gynecol | Genetic and non-genetic risk factors for pre-eclampsia: umbrella review of systematic reviews and meta-analyses of observational studies | 130 | NR |
| Lucaroni F | 2018 | J Matern Fetal Neonatal Med | Can risk be predicted? An umbrella systematic review of current risk prediction models for cardiovascular diseases, diabetes and hypertension | 14 | 360 |
| Machado MO | 2018 | BMC Med | The association of depression and all-cause and cause-specific mortality: an umbrella review of systematic reviews and meta-analyses | 24 | 246 |
| O'Sullivan JW | 2018 | BMJ | Prevalence and outcomes of incidental imaging findings: umbrella review | 20 | 240 |
| Parreira P | 2018 | Spine J | Risk factors for low back pain and sciatica: an umbrella review | 54 | 134 |
| Radua J | 2018 | World Psychiatry | What causes psychosis? An umbrella review of risk and protective factors | 55 | 683 |
| Bellou V | 2019 | Int J Tuberc Lung Dis | Elucidating the risk factors for chronic obstructive pulmonary disease: an umbrella review of meta-analyses | 11 | NR |
| Bouras E | 2019 | Gene | Gene promoter methylation and cancer: An umbrella review | 80 | 1323 |
| Fullana MA | 2019 | Psychol Med | Risk and protective factors for anxiety and obsessive-compulsive disorders: an umbrella review of systematic reviews and meta-analyses | 46 | 216 |
| Giannakou K | 2019 | PLOS One | Risk factors for gestational diabetes: An umbrella review of meta-analyses of observational studies | 61 | NR |
| Kim JY | 2019 | Lancet Psychiatry | Environmental risk factors and biomarkers for autism spectrum disorder: an umbrella review of the evidence | 119 | NR |
| Lucaroni F | 2019 | BMJ Open | Biomarkers for predicting spontaneous preterm birth: an umbrella systematic review | 16 | 674 |
| Piovani D | 2019 | Gastroenterology | Environmental Risk Factors for Inflammatory Bowel Diseases: An Umbrella Review of Meta-analyses | 53 | NR |
| Raglan O | 2019 | Int J Cancer | Risk factors for endometrial cancer: An umbrella review of the literature | 61 | 1354 |
| Tortella-Feliu M | 2019 | Neurosci Biobehav Rev | Risk factors for posttraumatic stress disorder: An umbrella review of systematic reviews and meta-analyses | 33 | 238 |
| Yang T | 2019 | Int J Cancer | Gene-environment interactions and colorectal cancer risk: An umbrella review of systematic reviews and meta-analyses of observational studies | 20 | NR |
| Zhou C | 2019 | Frontiers in Oncology | Prognostic Biomarkers for Gastric Cancer: An Umbrella Review of the Evidence | 74 | 1000 |
| Arias-de la Torre J | 2020 | J Med Internet Res | Relationship Between Depression and the Use of Mobile Technologies and Social Media Among Adolescents: Umbrella Review | 3 | NR |
| Belbasis L | 2020 | Eur J Epidemiol | Environmental factors, serum biomarkers and risk of atrial fibrillation: an exposure-wide umbrella review of meta-analyses | 51 | NR |
| Bonaccorsi | 2020 | International Journal of Environmental Research and Public Health | mpact of the Built Environment and the Neighborhood in Promoting the Physical Activity and the Healthy Aging in Older People: An Umbrella Review | 3 | 682 |
| Carvalho AF | 2020 | Translational Psychiatry | Evidence-based umbrella review of 162 peripheral biomarkers for major mental disorders | 110 | 359 |
| Gatti M | 2020 | European Journal of Internal Medicine | Assessing the association between fluoroquinolones and emerging adverse drug reactions raised by regulatory agencies: An umbrella review | 7 | NR |
| Grabovac I | 2020 | Clin Infect Dis | Human Immunodeficiency Virus Infection and Diverse Physical Health Outcomes: An Umbrella Review of Meta-analyses of Observational Studies | 20 | NR |
| Hossain MM | 2020 | Internation Journal of Social Psychiatry | Prevalence of mental disorders among people who are homeless: An umbrella review | 7 | NR |
| Hossain MM | 2020 | Asian J Psychiatr | Prevalence of comorbid psychiatric disorders among people with autism spectrum disorder: An umbrella review of systematic reviews and meta-analyses | 12 | NR |
| Hossain MM | 2020 | Psychiatry Res | Prevalence of mental disorders in South Asia: An umbrella review of systematic reviews and meta-analyses | 11 | NR |
| Hutchens BF | 2020 | J Midwifery Womens Health | Risk Factors for Postpartum Depression: An Umbrella Review | 21 | 1143 |
| Kelly, M. M. | 2020 | J Am Assoc Nurse Pract | The Influence of preterm birth beyond infancy: Umbrella review of outcomes of adolescents and adults born preterm | 9 | NR |
| Kelly, M. M. | 2020 | J Pediatr Health Care | Umbrella Review of School Age Health Outcomes of Preterm Birth Survivors | 21 | NR |
| Kim, J. H. | 2020 | Lancet Psychiatry | Environmental risk factors, protective factors, and peripheral biomarkers for ADHD: an umbrella review | NR | NR |
| Li L | 2020 | BMJ Open | Association between H. pylori infection and health Outcomes: an umbrella review of systematic reviews and meta-analyses | 60 | 1239 |
| Lin X, Xia L | 2020 | Cancer Medicine | Sarcopenia and adverse health-related outcomes: An umbrella review of meta-analyses of observational studies | 30 | NR |
| Marco, Solmi | 2020 | Neuroscience and biobehavioral reviews | Risk and protective factors for mental disorders with onset in childhood/adolescence: an umbrella review of published meta-analyses of observational longitudinal studies | 10 | 192 |
| Martinez-Calderon, J. | 2020 | Clin J Pain | Which Psychological Factors Are Involved in the Onset and/or Persistence of Musculoskeletal Pain? An Umbrella Review of Systematic Reviews and Meta-Analyses of Prospective Cohort Studies | NR | 286 |
| Mohammed, S. H. | 2020 | BMC Pediatrics | The state of child nutrition in Ethiopia: an umbrella review of systematic review and meta-analysis reports | 9 | 255 |
| Obaid, M. | 2020 | BMJ Open | Can we prevent poststroke cognitive impairment? An umbrella review of risk factors and treatments | NR | NR |
| Okoth, K. | 2020 | Bmj | Association between the reproductive health of young women and cardiovascular disease in later life: umbrella review | 24 | NR |
| Palmer Kelly, E. | 2020 | Surg Oncol | The role of religion and spirituality in cancer care: An umbrella review of the literature | 6 | NR |
| Pelletier, R. | 2020 | Endocrinol Diabetes Metab | Adverse events associated with sodium glucose co-transporter 2 inhibitors: an overview of quantitative systematic reviews | 8 | NR |
| Peruzzi, M. | 2020 | Curr Emerg Hosp Med Rep | Vaping Cardiovascular Health Risks: an Updated Umbrella Review | NR | 183 |
| Rahmanian, V. | 2020 | J Family Med Prim Care | Seroprevalence of toxoplasma gondii infection: An umbrella review of updated systematic reviews and meta-analyses | NR | NR |
| Smith J | 2020 | Ageing Res Rev | Telomere length and health outcomes: An umbrella review of systematic reviews and meta-analyses of observational studies | 4 | 167 |
| Solmi M | 2020 | Braz J Psychiatry | Risk factors for eating disorders: an umbrella review of published meta-analyses | 9 | NR |
| Solmi M | 2020 | Journal of Affective Disorders | Factors Associated With Loneliness: An Umbrella Review Of Observational Studies | NR | 795 |
| Townsend R | 2020 | BJOG | Prediction of stillbirth: an umbrella review of evaluation of prognostic variables | NR | NR |
| Tsiros MD | 2020 | Obesity Reviews | Obesity, the new childhood disability? An umbrella review on the association between adiposity and physical function | NR | NR |
| van der Burg NC | 2020 | Molecular Psychiatry | The genetics of drug-related movement disorders an umbrella review of meta-analyses | 15 | NR |
| Wang Y | 2020 | Front. Oncol | Prognostic Biomarkers for Pancreatic Ductal Adenocarcinoma: An Umbrella Review | 41 | NR |
| Xia L | 2020 | Cancer Medicine | Sarcopenia and adverse health-related outcomes: An umbrella review of meta-analyses of observational studies | 30 | NR |
| Xu W | 2020 | BMC Medicine | Risk factors and risk prediction models for colorectal cancer metastasis and recurrence: an umbrella review of systematic reviews and meta-analyses of observational studies | 81 | NR |
| Zhang X | 2020 | Cancer Medicine | Non-genetic biomarkers and colorectal cancer risk: Umbrella review and evidence triangulation | NR | NR |
| Abou Ghayda R | 2021 | European Review for Medical and Pharmacological Sciences | Body mass index and mortality in patients with cardiovascular disease: an umbrella review of meta-analyses | NR | NR |
| Avsar TS | 2021 | BMC Pregnancy and Chidbirth | Health outcomes of smoking during pregnancy and the postpartum period: an umbrella review | 11 | NR |
| Balante | 2021 | International Journal of Nursing Studies | How does culture influence work experience in a foreign country? An umbrella review of the cultural challenges faced by internationally educated nurses | 1 | NR |
| Bertelsen | 2021 | Basic Clin Pharmacol Toxicol | Are older adults insufficiently included in clinical trials?-An umbrella review | 3 | NR |
| Blume K | 2021 | Health Services Research | How does culture influence work experience in a foreign country? An umbrella review of the cultural challenges faced by internationally educated nurses | 2 | 201 |
| Bramley P | 2021 | International Journal of Surgery | Risk factors for postoperative delirium: An umbrella review of systematic reviews | 8 | NR |
| Burns CJ | 2021 | International Archives of Occupational and Environmental Health | Cancer and occupational exposure to pesticides: an umbrella review | NR | NR |
| Fan J | 2021 | International Journal of Environment Research and Public Health | An Umbrella Review of the Work and Health Impacts of Working in an Epidemic/Pandemic Environment | NR | NR |
| Farazi M | 2021 | Critical Reviews in Food Science and Nutrition | Dietary inflammatory index and the risk of non-communicable chronic disease and mortality: an umbrella review of meta-analyses of observational studies | 11 | 127 |
| Iqbal, S. | 2021 | International Journal of Environmental Health Research | Maternal pesticide exposure and its relation to childhood cancer: an umbrella review of meta-analyses | 19 | NR |
| Kim, M. S. | 2021 | Eur Heart J | ssociation between adiposity and cardiovascular outcomes: an umbrella review and meta-analysis of observational and Mendelian randomization studies | NR | 501 |
| Lee KS | 2021 | J Korean Med Sci | Environmental and Genetic Risk Factors of Congenital Anomalies: an Umbrella Review of Systematic Reviews and Meta-Analyses | 66 | NR |
| Li W | 2021 | Ophthalmic Res | Non-ocular influence factors for primary glaucoma：an umbrella review of meta-analysis | 22 | NR |
| Lopez-Leon, S. | 2021 | Neuroscience and Biobehavioral Reviews | Molecular genetics of substance use disorders: An umbrella review | 85 | 58 |
| Magnavita, N. | 2021 | Int J Environ Res Public Health | SARS/MERS/SARS-CoV-2 Outbreaks and Burnout Syndrome among Healthcare Workers. An Umbrella Systematic Review | 3 | NR |
| Mjaess, G. | 2021 | Acta Oncol | Prognostic role of neutrophil-to-lymphocyte ratio (NLR) in urological tumors: an umbrella review of evidence from systematic reviews and meta-analyses | 28 | NR |
| Paraskevi, L. | 2021 | Mater Sociomed | Stress and Anxiety Levels in Couples who Undergo Fertility Treatment: a Review of Systematic Reviews | NR | NR |
| Pelletier, R. | 2021 | Ther Adv Drug Saf | The association of sodium-glucose cotransporter 2 inhibitors with cancer: An overview of quantitative systematic reviews | 47 | NR |
| Pittara, T. | 2021 | Bjog | Pre-eclampsia and long-term health outcomes for mother and infant: an umbrella review | 21 | NR |
| Poveda-Moral, S. | 2021 | Worldviews Evid Based Nurs | Barriers to Advance Care Planning Implementation in Health care: An Umbrella Review with Implications for Evidence-Based Practice | NR | NR |
| Román-Gálvez R. M. | 2021 | Int J Environ Res Public Health | Prevalence of Intimate Partner Violence in Pregnancy: An Umbrella Review | 5 | NR |
| Rydzewska E | 2021 | The British Journal of Psychiatry | Umbrella systematic review of systematic reviews and meta-analyses on comorbid physical conditions in people with autism spectrum disorder | 12 | NR |
| Sahebi A | 2021 | Prog Neuropsychopharmacol Biol Psychiatry | The prevalence of anxiety and depression among healthcare workers during the COVID-19 pandemic: An umbrella review of meta-analyses | 7 | 108 |
| Sahle BW | 2021 | European Child & Adolescent Psychiatry | The association between adverse childhood experiences and common mental disorders and suicidality: an umbrella review of systematic reviews and meta-analyses | 68 | NR |
| Shams Vahdati | 2021 | Egypt J Neurol Psychiatry Neurosurg | Neurological manifestations of COVID-19 infection: an umbrella review | 12 | NR |
| Solmi M | 2021 | Neuroscience and Biobehavioral Reviews | Risk and protective factors for alcohol and tobacco related disorders: An umbrella review of observational studies | 12 | NR |
| Solmi M | 2021 | Neuroscience and Biobehavioral Reviews | Risk and protective factors for mental disorders with onset in childhood/adolescence: An umbrella review of published meta-analyses of observational longitudinal studies | 10 | 192 |
| Solmi M | 2021 | Neuroscience and Biobehavioral Reviews | Risk and protective factors for cannabis, cocaine, and opioid use disorders: An umbrella review of meta-analyses of observational studies | 5 | 180 |
| Thang VN | 2021 | Pediatr Dent | Risk Factors for Early Childhood Caries: An Umbrella Review | 8 | 35 |
| Trott M | 2021 | Wien Klin Wochenschr | Hearing impairment and diverse health outcomes : An umbrella review of meta-analyses of observational studies | NR | NR |
| Weir, N. M. | 2021 | Res Social Adm Pharm | Development of a primary care pharmacy outcomes framework: An umbrella literature review | 1 | 14 |
| Zhang TN | 2021 | Front. Cardiovasc | Environmental Risk Factors and Congenital Heart Disease: An Umbrella Review of 165 Systematic Reviews and Meta-Analyses With More Than 120 Million Participants | NR | 1088 |

Abbreviations: MA – meta-analysis, No. – number, NR – not reported

## **S5 Table** Details of method used for certainty and methodological quality assessment in included umbrella reviews

| **First author** | **publication year** | **Method used** | |
| --- | --- | --- | --- |
|  |  | **Certainty of evidence assessment** | **Methodological quality assessment** |
| Belbasis L | 2015 | Criteria for credibility assessment | Not performed |
| Apostolo J | 2016 | Not performed | JBI critical appraisal checklist |
| Belbasis L | 2016 | Criteria for credibility assessment | Not performed |
| Belbasis L | 2016 | Criteria for credibility assessment | Not performed |
| Belbasis L | 2016 | Criteria for credibility assessment | Not performed |
| Bellou V | 2016 | Criteria for credibility assessment | Not performed |
| Campbell JM | 2016 | Not performed | Joanna Briggs Institute Umbrella Review Assessment and Review of Information (JBI-URARI) appraisal tool for systematic reviews |
| Campbell JM | 2016 | Not performed | JBI critical appraisal checklist |
| Carvalho AF | 2016 | Criteria for credibility assessment | Not performed |
| Bellou V | 2017 | Criteria for credibility assessment | Not performed |
| Bortolato B | 2017 | Criteria for credibility assessment | AMSTAR |
| Campbell JM | 2017 | Not performed | Joanna Briggs Institute Umbrella Review Assessment and Review of Information (JBI-URARI) appraisal tool for systematic reviews |
| Belbasis L | 2018 | Criteria for credibility assessment | Not performed |
| Belbasis L | 2018 | Criteria for credibility assessment | Not performed |
| Bellou V | 2018 | Criteria for credibility assessment | Not performed |
| Cupp, M. | 2018 | Criteria for credibility assessment | AMSTAR 2 |
| Giannakou K | 2018 | Criteria for credibility assessment | Newcastle Ottawa Scale |
| Lucaroni F | 2018 | Not performed | AMSTAR |
| Machado MO | 2018 | Criteria for credibility assessment | AMSTAR |
| O'Sullivan JW | 2018 | Not performed | AMSTAR |
| Parreira P | 2018 | Not performed | AMSTAR |
| Radua J | 2018 | Criteria for credibility assessment | AMSTAR |
| Bellou V | 2019 | Criteria for credibility assessment | Not performed |
| Bouras E | 2019 | Criteria for credibility assessment | AMSTAR |
| Fullana MA | 2019 | Criteria for credibility assessment | Not performed |
| Giannakou K | 2019 | Criteria for credibility assessment | AMSTAR |
| Kim JY | 2019 | Criteria for credibility assessment | Not performed |
| Lucaroni F | 2019 | Not performed | AMSTAR |
| Piovani D | 2019 | Criteria for credibility assessment | AMSTAR 2 |
| Raglan O | 2019 | Criteria for credibility assessment | AMSTAR |
| Tortella-Feliu M | 2019 | Criteria for credibility assessment | Not performed |
| Yang T | 2019 | Criteria for credibility assessment | Not performed |
| Zhou C | 2019 | Criteria for credibility assessment | AMSTAR 2 |
| Arias-de la Torre J | 2020 | Not performed | Authors used their own criteria |
| Belbasis L | 2020 | Criteria for credibility assessment | Not performed |
| Bonaccorsi | 2020 | Not performed | Not performed |
| Carvalho AF | 2020 | Criteria for credibility assessment | AMSTAR 2 |
| Gatti | 2020 | Criteria for credibility assessment | AMSTAR 2 |
| Grabovac I | 2020 | Criteria for credibility assessment | AMSTAR 2 |
| Hossain (3) | 2020 | Not performed | JBI critical appraisal checklist |
| Hossain MM | 2020 | Not performed | JBI critical appraisal checklist |
| Hossain MM | 2020 | Not performed | JBI critical appraisal checklist |
| Hutchens BF | 2020 | Not performed | AMSTAR |
| Kelly, M. M. | 2020 | Not performed | JBI critical appraisal checklist |
| Kelly, M. M. | 2020 | Not performed | JBI critical appraisal checklist |
| Kim, J. H. | 2020 | Criteria for credibility assessment | AMSTAR 2 |
| Li L | 2020 | GRADE approach | AMSTAR 2 |
| Lin X, Xia L | 2020 | GRADE approach | AMSTAR |
| Marco, Solmi | 2020 | Criteria for credibility assessment | AMSTAR 2 |
| Martinez-Calderon, J. | 2020 | Not performed | AMSTAR 2 |
| Mohammed, S. H. | 2020 | Not performed | AMSTAR |
| Obaid, M. | 2020 | GRADE approach | AMSTAR 2 |
| Okoth, K. | 2020 | Not performed | AMSTAR 2 |
| Palmer Kelly, E. | 2020 | Not performed | Not performed |
| Pelletier, R. | 2020 | Not performed | AMSTAR 2 |
| Peruzzi, M. | 2020 | Not performed | Oxman-Guyatt index |
| Rahmanian, V. | 2020 | Not performed | AMSTAR |
| Smith J | 2020 | Not performed | AMSTAR |
| Solmi M | 2020 | Criteria for credibility assessment | AMSTAR 2 |
| Solmi M | 2020 | Criteria for credibility assessment | AMSTAR 2 |
| Townsend R | 2020 | Criteria for credibility assessment | AMSTAR |
| Tsiros MD | 2020 | Not performed | JBI critical appraisal checklist |
| van der Burg NC | 2020 | Not performed | AMSTAR |
| Wang Y | 2020 | Criteria for credibility assessment | AMSTAR 2 |
| Xia L | 2020 | GRADE approach | AMSTAR |
| Xu W | 2020 | Criteria for credibility assessment | AMSTAR 2 |
| Zhang X | 2020 | Criteria for credibility assessment | Not performed |
| Abou Ghayda R | 2021 | Criteria for credibility assessment | Not performed |
| Avsar TS | 2021 | Authors used their own criteria (applying a categorization of the strength of evidence of different variables based on the share of significant results and the number of literature reviews) | A tool developed from the Centre for Reviews and Dissemination (CRD) checklist |
| Balante | 2021 | Not performed | ROBIS |
| Bertelsen | 2021 | Not performed | AMSTAR |
| Blume K | 2021 | Authors used their own criteria (applying a categorization of the strength of evidence of different variables based on the share of significant results and the number of literature reviews) | AMSTAR 2 |
| Bramley P | 2021 | Not performed | ROBIS |
| Burns CJ | 2021 | Not performed | Not performed |
| Fan J | 2021 | Not performed | JBI critical appraisal checklist |
| Farazi M | 2021 | GRADE approach | AMSTAR 2 |
| Iqbal, S. | 2021 | Not performed | Not performed |
| Kim, M. S. | 2021 | GRADE approach | AMSTAR 2 |
| Lee KS | 2021 | GRADE approach | AMSTAR 2 |
| Li W | 2021 | Criteria for credibility assessment | AMSTAR 2 |
| Lopez-Leon, S. | 2021 | Criteria for credibility assessment | Not performed |
| Magnavita, N. | 2021 | Not performed | Not performed |
| Mjaess, G. | 2021 | GRADE approach | AMSTAR 2 |
| Paraskevi, L. | 2021 | Not performed | ROBIS |
| Pelletier, R. | 2021 | Not performed | AMSTAR 2 |
| Pittara, T. | 2021 | Criteria for credibility assessment | AMSTAR |
| Poveda-Moral, S. | 2021 | Not performed | JBI critical appraisal checklist |
| Román-Gálvez R. M. | 2021 | Not performed | AMSTAR 2 |
| Rydzewska E | 2021 | Not performed | JBI critical appraisal checklist |
| Sahebi A | 2021 | Not performed | AMSTAR 2 |
| Sahle BW | 2021 | Not performed | AMSTAR 2 |
| Shams Vahdati | 2021 | Not performed | AMSTAR 2 |
| Solmi M | 2021 | Criteria for credibility assessment | AMSTAR 2 |
| Solmi M | 2021 | Criteria for credibility assessment | AMSTAR 2 |
| Solmi M | 2021 | Criteria for credibility assessment | AMSTAR 2 |
| Thang VN | 2021 | Not performed | AMSTAR 2 |
| Trott M | 2021 | Using GRADE and criteria for credibility assessment separately for meta-analyses of epidemiologic evidence and meta-analyses of RCTs | AMSTAR 2 |
| Weir, N. M. | 2021 | Not performed | Not performed |
| Zhang TN | 2021 | Criteria for credibility assessment | AMSTAR 2 |

Abbreviation: AMSTAR - A Measurement Tool to Assess Systematic Reviews, GRADE - The Grading of Recommendations Assessment, Development and Evaluation, JBI - Joanna Briggs Institute, RCTs – Randomized controlled trials, ROBIS - A Risk of Bias Assessment Tool for Systematic Reviews

## **S6 Table** Details of criteria for credibility assessment used in included URs

| **Details of criteria** | **Criteria for credibility assessment of each study** | | | | | |
| --- | --- | --- | --- | --- | --- | --- |
| group | 1 | 2 | **3** | 4 | others | no detail |
| Number of studies (%) | 10 (28.6) | 3 (8.6) | 2 (5.7) | 2 (5.7) | 15 (42.8) | 3 (8.6) |
| Median JIF | 8.4 [6.7-9.0] | 8.5 [6.1-49.5] | 6.9 [6.6-7.2] | 7.1 [6.5-7.7] | 6.2 [4.1-8.9] | 3.8 [2.9-6.2] |
| **1. Number of categories** | 5 | 5 | 5 | 4 | [1 – 5] | 5 |
| **2. Details of each category** |  |  |  |  |  |  |
| **2.1 Convincing/class I** |  |  |  |  |  |  |
| Number of cases | >1000 cases | >1000 cases | >1000 cases (or >20,000 participants for continuous outcomes) | >1000 cases | - Not specified - >1000 cases (or >20,000 participants for continuous outcomes) |  |
| P-value | p <10^−6^ | p < 10^−6^ | p <10^−6^ | p < 10^−6^ | p<0.005 to p <10^−6^ |  |
| 95% prediction interval excluded null | Prediction intervals not including the null value | Prediction intervals not including the null value | Prediction intervals not including the null value | Prediction intervals not including the null value | - Not specified - Prediction intervals not including the null value |  |
| Heterogeneity | I^2^ < 50% | I^2^ < 50% | I^2^ < 50% | I^2^ < 50% | - Not specified - I^2^ < 50% |  |
| No evidence of small-study effects | No evidence of small-study effects | no evidence of small study effects | no evidence of small study effects (p > 0.10) | no evidence of small study effects | - Not specified - no evidence of small study effects (p > 0.10, for Egger's test) |  |
| No evidence of excess significance bias | No evidence of excess of significance bias | No evidence of excess of significance bias | No evidence of excess of significance bias | No evidence of excess of significance bias | - Not specified - No evidence of excess of significance bias |  |
| Largest study with statistically significant effect | Largest study nominally significant (p < 0.05) |  | Largest study nominally significant (p < 0.05) |  | - Not specified - Largest study nominally significant (p < 0.05) |  |
| Credibility ceiling |  |  |  |  | - survive the 10% credibility ceiling test |  |
| **2.2 Highly suggestive/class II** |  |  |  |  |  |  |
| Number of cases | >1000 cases | >1000 cases | >1000 cases (or more than 20,000 participants for continuous outcomes) | >1000 cases | - Not specified - >1000 cases (or >20,000 participants for continuous outcomes) |  |
| P-value | p <10^−6^ | p <10^−6^ | p<10^-3^ | p <10^−6^ | - Not specified to p <10^−6^ |  |
| 95% prediction interval excluded null |  |  |  |  | - Not specified |  |
| Heterogeneity |  |  |  |  | - Not specified |  |
| No evidence of small-study effects | Largest study nominally significant (p < 0.05) | Largest study nominally significant (p < 0.05) | Largest study nominally significant (p < 0.05) |  | - Not specified |  |
| No evidence of excess significance bias |  |  |  |  | - Not specified |  |
| Largest study with statistically significant effect |  |  |  |  | - Not specified - Largest study nominally significant (p < 0.05) |  |
| **2.3 Suggestive/class III** |  |  |  |  |  |  |
| Number of cases | >1000 cases | >1000 cases | >1000 cases (or more than 20,000 participants for continuous outcomes) | >1000 cases | - Not specified - >1000 cases (or >20,000 participants for continuous outcomes) |  |
| P-value | p <10^−3^ | p <10^−3^ | p<10^-2^ | p <10^−3^ | p <10^−2^ to p <10^−3^ |  |
| Heterogeneity |  |  |  |  | - Not specified |  |
| **2.4 Weak/class IV** |  |  |  |  |  |  |
| P-value | p < 0.05 | p < 0.05 | p < 0.05 | p < 0.05 | p < 0.05 |  |
| **2.5 Non-significant** |  |  |  |  |  |  |
| P-value | p > 0.05 |  | p >0.05 |  | p >0.05 |  |

## **S2 file:** Protocol

# Methodological approaches for assessing certainty of the evidence in umbrella reviews:

# a systematic review

*Saranrat Sadoyu, Nathorn Chaiyakunapruk, Sobhon Paengtrai, Nontaporn Punrum, Nai Ming Lai, Ammarin Thakkinstian, Surachat Ngorsuraches, Kaniz A. Tanni, Mukdarut Bangpan*

# Citation

## Saranrat Sadoyu, Nathorn Chaiyakunapruk, Sobhon Paengtrai, Nontaporn Punrum, Nai Ming Lai, Ammarin Thakkinstian, Surachat Ngorsuraches, Kaniz A. Tanni, Mukdarut Bangpan.

Methodological approaches for assessing certainty of the evidence in umbrella reviews: a systematic review. PROSPERO 2020 CRD42020203273 Available from: <https://www.crd.york.ac.uk/prospero/display_record.php?ID=CRD42020203273>

Review question

What approaches have been used to assess the certainty of the evidence in the umbrella review? How many of umbrella review of meta-analyses assessed the certainty of the evidence?

To identify and describe the methodological approaches for assessing the certainty of the evidence in umbrella reviews of meta-analyses.

# Searches

We will systematically search the following bibliographical databases: PubMed, Embase, and The Cochrane Library. We will restrict the search for the latest ten years (from May 2010 to May 2020) to obtain contemporary evidence.

The search strategy will include the keywords ‘umbrella review’. No language restriction will be applied for searching. Manual searches of the reference lists of eligible articles will also be performed to identify additional studies that may not have been retrieved through search strategies.

Titles and abstracts of all retrieved articles will be screened by two reviewers independently (SS and SP). The full text of potentially eligible articles will be read thoroughly to choose those that fulfil the eligibility criteria. Any disagreements will be resolved with a third reviewer (NC) via consensus.

# Types of study to be included

Eligibility criteria for the searches: Inclusion criteria:

1. The umbrella reviews that included systematic reviews with meta-analysis. Exclusion criteria

1. Handbooks, guidelines, commentaries, editorials, letters, expert opinion, scoping review, and methodological studies
2. Conference abstracts, conference proceedings, and abstracts for poster presentation
3. Umbrella reviews that further performed a network meta-analysis
4. Protocols of umbrella reviews

Two reviewers (SS and SP) will independently review the title, abstracts and full text for their potential inclusion against the eligibility criteria. Any disagreement will be resolved by discussion with a third reviewer

(NC).

# Condition or domain being studied

Umbrella review of meta-analyses which we will define an ‘umbrella review’ as: The review that designed to summarize the evidence from multiple systematic reviews and meta-analyses that were labelled as ‘umbrella review’ in the title or abstract of the article.

# Participants/population

Any (no restriction)

# Intervention(s), exposure(s)

Our interested factors are the methodological approach used for assessing the certainty (or quality) of the evidence in umbrella reviews of meta-analyses.

# Comparator(s)/control

None

# Main outcome(s)

Methods used for assessing the certainty of the evidence in an umbrella review of meta-analyses.

## * Measures of effect

Frequency and proportion of studies assessing the certainty of the evidence

# Additional outcome(s)

The extent (frequency) of the umbrella reviews of meta-analysis that assessed the certainty of the evidence.

Timing and effect measures Not applicable

## * Measures of effect

Frequency and proportion of studies using a certain method

# Data extraction (selection and coding)

A customized data extraction form table will be developed using Excel. At least two reviewers (SS, NP, SN, KT, and SP) independently extracted the data. Any disagreements will be resolved by discussion. If disagreements could not be resolved, the same third reviewer (NC) will be consulted.

From each eligible article, methods will be extracted as reported irrespective of the section of the article in which they were described (e.g., methods, results, footers of tables, discussion, or supplementary files). Assessment of the certainty of evidence will be defined as an evaluation of the totality or strength of the evidence including the GRADE approach, criteria for credibility assessment, and other approaches used to grade the overall body of the evidence. Specifically, the following information will be extracted from each included study:

Study/author characteristics:

1. The first author
2. Year of publication
3. Title
4. Journal published
5. Number of meta-analyses included
6. Study design of the primary studies included in the meta-analysis (e.g. RCTs, clinical trials, observational studies)
7. Characteristics of participants, interventions/exposures, comparators, outcomes examined Methodological characteristics:
8. Method/Approach/Tool or specific tool used for assessing the certainty of the evidence in the umbrella review (i.e. GRADE approach, criteria for credibility assessment, and NutriGrade).
9. Description of the methods
   1. Domains and items
   2. How to assess
   3. How to grade each item, domain, and overall
10. Method/Approach/Tool used to assess the methodological quality/risk of bias of the included systematic reviews

# Risk of bias (quality) assessment

Risk of bias assessment will not be considered in this review as we are not interested in the potential bias of included studies or drawing inferences from them. The focus of this systematic review is to identify and describe methods.

# Strategy for data synthesis

We will perform a narrative synthesis of the methodological approaches used for assessing the certainty of the evidence in the umbrella review. Tables and graphs will be used to summarize data. We will conduct a descriptive analysis using frequencies to summarize the variables collected.

# Analysis of subgroups or subsets

As there is not a gold standard for assessing the certainty of the evidence in the umbrella review, we will provide overall results and the results separately for each type umbrella review that classified using the type of included articles, as follows;

1. Question types: umbrella review will be categorized according to the question types of the included meta- analyses (1-3), as follows;
   1. Intervention or therapy (Prevention and treatment): This type refers to questions of prevention/treatment in order to achieve a specific outcome. These questions may include drugs, surgical intervention, change in diet, counselling, and modifiable risk factors.
   2. Non-intervention/therapy: The umbrella reviews that included meta-analyses performing based on review questions including;

- Diagnosis or diagnostic test
- Prognosis or prediction
- Etiology
- Prevalence and incidence

Furthermore, these umbrella reviews will be categorized into 3 groups regarding the study design of primary studies that included in the meta-analyses; meta-analyses of experimental studies, meta-analyses of non- experimental studies, and meta-analyses of the mixed study designs.

1. Impact of the journal: Umbrella review will be classified into high and lower impact groups based on the total citations in 2019 of their published journals. We modified the criteria from the approach used in Bala et al, 2013(4).

Contact details for further information Professor Nathorn Chaiyakunapruk [Nathorn.Chaiyakunapruk@utah.edu](mailto:Nathorn.Chaiyakunapruk@utah.edu)

# Organisational affiliation of the review

University of Utah

# Review team members and their organisational affiliations

Ms Saranrat Sadoyu. Chulalongkorn University Professor Nathorn Chaiyakunapruk. University of Utah Mr Sobhon Paengtrai. Chiangmai University

Ms Nontaporn Punrum. Chiangmai University, Professor Nai Ming Lai. Taylor's University

Professor Ammarin Thakkinstian. Ramathibodi Hospital, Mahidol University Dr Surachat Ngorsuraches. Harrison School of Pharmacy, Auburn university Ms Kaniz A. Tanni. Harrison School of Pharmacy, Auburn University

Assistant/Associate Professor Mukdarut Bangpan. UCL Social Research Institute

# Type and method of review

Intervention, Methodology, Narrative synthesis, Review of reviews, Systematic review

# Anticipated or actual start date

01 July 2020

# Anticipated completion date

30 September 2020

# Funding sources/sponsors

None

# Conflicts of interest Language

English

# Country

England, Malaysia, United States of America

# Stage of review

Review Ongoing

# Subject index terms status

Subject indexing assigned by CRD

# Subject index terms

Humans; Research Design

# Date of registration in PROSPERO

22 September 2020

# Date of first submission

| 07 August 2020 |  |
| --- | --- |
